# Indoor Localization Using Uncooperative Wi-Fi Access Points

**DOI:** 10.3390/s22083091

**Published:** 2022-04-18

**Authors:** Berthold K. P. Horn

**Affiliations:** Department of Electrical Engineering and Computer Science, Massachusetts Institute of Technology, Cambridge, MA 02139, USA; bkph@csail.mit.edu

**Keywords:** one-sided RTT, two-sided RTT, indoor position, indoor location, relative permittivity, fine timing measurement, round-trip time, FTM, uncooperative AP, IEEE 802.11-2016, IEEE 802.11mc, Bayesian grid, observation model, transition model

## Abstract

Indoor localization using fine time measurement (FTM) round-trip time (RTT) with respect to cooperating Wi-Fi access points (APs) has been shown to work well and provide 1–2 m accuracy in both 2D and 3D applications. This approach depends on APs implementing the IEEE 802.11-2016 (also known as IEEE 802.11mc) Wi-Fi standard (“two-sided” RTT). Unfortunately, the penetration of this Wi-Fi protocol has been slower than anticipated, perhaps because APs tend not to be upgraded as often as other kinds of electronics, in particular in large institutions—where they would be most useful. Recently, Google released Android 12, which also supports an alternative “one-sided” RTT method that will work with legacy APs as well. This method cannot subtract out the “turn-around” time of the signal, and so, produces distance estimates that have much larger offsets than those seen with two-sided RTT—and the results are somewhat less accurate. At the same time, this method makes possible distance measurements for many APs that previously could not be used. This increased accessibility can compensate for the decreased accuracy of individual measurements. We demonstrate here indoor localization using *one-sided* RTT with respect to legacy APs that do not support IEEE 802.11-2016. The accuracy achieved is 3–4 m in cluttered environments with few line-of-sight readings (and using only 20 MHz bandwidths). This is not as good as for *two-sided* RTT, where 1–2 m accuracy has been achieved (using 80 MHz bandwidths), but adequate for many applications A wider Wi-Fi channel bandwidth would increase the accuracy further. As before, Bayesian grid update is the preferred method for determining position and positional accuracy, but the observation model now is different from that for two-sided RTT. As with two-sided RTT, the probability of an RTT measurement below the true distance is very low, but, in the other direction, the range of measurements for a given distance can be much wider (up to well over twice the actual distance). We describe methods for formulating useful observation models. As with two-sided RTT, the offset or bias in distance measurements has to be subtracted from the reported measurements. One difference is that here, the offsets are large (typically in the 2400–2700 m range) because of the “turn-around time” of roughly 16 μs (i.e., about two orders of magnitude larger than the time of flight one is attempting to measure). We describe methods for estimating these offsets and for minimizing the effort required to do so when setting up an installation with many APs.

## 1. Background

There has been considerable work on methods for localizing position indoors where GPS cannot be used or where ordinary GPS is not accurate enough [1,2,3,4,5,6,7,8,9,10,11,12]. The IEEE 802.11-2016 (also known as IEEE 802.11mc) Wi-Fi standard provides a protocol for an initiator (cell phone) to estimate its distance from a responder (Wi-Fi AP) [13,14,15,16,17,18,19,20,21,22,23,24,25,26].

Actually, what is reported is half of the round-trip time (RTT) of an RF signal, minus the turn-around time, multiplied by the speed of light. Methods based on this have been implemented and reported [15,27,28] and produce good accuracy (1–2 m) with very little setup effort—other than needing to know the locations of the APs.

While the 802.11-2016 protocol was approved in 2016 and the first Wi-Fi chips supporting it have been available since at least 2018, there are still relatively few installed Wi-Fi APs that advertise support for it in the beacon frame. Interestingly, the number of APs that respond to ranging requests *has* gone up quite a bit, yet the number that *advertise* this capability remains low. It is not known why some devices do not advertise this capability, if they have it, but it is somewhat more awkward to utilize them for ranging with the API provided for two-sided RTT ranging. In any case, at this point, indoor localization using this approach often requires installing APs that *do* support the protocol—which somewhat defeats the purpose of piggy-backing on existing Wi-Fi infrastructure.

## 2. Brief Review: Two-Sided FTM RTT

Two-sided FTM RTT has the “initiator” (cell phone) send out an RTT request to the “responder” (Wi-Fi AP), which then sends a specified number of messages to the initiator and waits for their acknowledgment. Importantly, the responder keeps track of when each message was sent ($t_{1}$) and when it was acknowledged ($t_{4}$), as measured by its clock. The timing information for one exchange ($t_{1}$ and $t_{4}$) is sent to the initiator in the message starting the *next* exchange. The initiator similarly keeps track of when it received the message ($t_{2}$) and when it acknowledged it ($t_{3}$), as measured by its own clock. The round-trip time (RTT) of the signal can then be easily calculated $\left(\left(t_{4}-t_{1}\right)-\left(t_{3}-t_{2}\right)\right)$. The two clocks need *not* be synchronized since the result is based on the differences between times measured by the *same* clocks. Note that the timing of the last exchange cannot be determined since the initiator does not receive the $t_{1}$ and $t_{4}$ corresponding to the last exchange. Hence, with *N* such exchanges in a burst, (N−1) measurements can be taken. By the way, typically, the “turn-around time” ($t_{3}-t_{2}$) is much longer than the signal “time of flight” (RTT), so it must be measured carefully.

The accuracy of the result depends roughly inversely on the bandwidth of the signal. Currently, Wi-Fi initiators supporting FTM RTT are limited to 20, 40, and 80 MHz. (Some Wi-Fi APs now support a bandwidth of 160 MHz as well, but smart phones do not yet). A complex signal with an 80 MHz bandwidth needs to have both I and Q (real and imaginary parts) sampled every 12.5 nsec. This corresponds to 3.75 m in round-trip distance or 1.875 m in one-way distance. This coarse quantization may appear to limit how accurately distances can be measured.

Fortunately, proprietary methods for interpolating the time of onset of the signal can provide estimates of arrival time quantized more finely than this, provided the signal-to-noise ratio (SNR) is large enough [25]. The Cramér–Rao lower bound (CRLB)—the theoretical lower bound for the standard deviation of the error in distance—is given by

(1) $$\sigma_{d}=\frac{1}{2\sqrt{2}\pi }\frac{1}{\sqrt{\mathrm{SNR}}}\frac{c}{B}$$

where *c* is the speed of light, *B* is the bandwidth in Hertz, and SNR is the signal-to-noise ratio (not in dB) [29,30,31]. For a bandwidth of 80 MHz (as in IEEE 802.11ac) and an SNR of 10, the CRLB comes to $\sigma_{d}=0.13\;m$, which is considerably smaller than the error observed in practice—which is more like 1 to 2 m. It is a bit of a puzzle why the actual error is considerably higher than the theoretical CRLB.

As expected, under line of sight (LOS) conditions, RTT measurements vary directly in step with distance (e.g., the best-fit line of RTT measurement versus actual distance has slope 1—see, e.g., Figure 4 in [27]). However, RTT measurements do have an offset, or bias, that depends on the equipment on the ends of the communication channel. The offset can be quite small (less than a meter) for some combinations (e.g., Google Pixel as the initiator and Google Wi-Fi as the responder). It can also be several meters in other cases (e.g., Compulab WILD as the responder). The offset can be different in different frequency ranges. For the best results, this offset needs to be determined and subtracted from the measurements.

RTT measurements (once corrected for offset) are good estimates of distance when there is a clear LOS and no obstacles in the first Fresnel zone between the initiator and the responder. This ideal is approximated in a large stadium or auditorium [15]. Inside most buildings, on the other hand, RTT (half of the round trip time times the speed of light) measurements should *not* be thought of simply as “distance”.

One reason is that RF signals are slowed down when they pass through objects. Many building materials (concrete, dry wall, wood, plaster, glass) have a high relative permittivity (2–20), and the speed of the signal is inversely proportional to the square root of the relative permittivity. Furthermore, if the direct path between the initiator and responder is blocked (such as by a metal wall), then reflections from outside the LOS come into play (multi-path). In both cases, the RTT measurement is greater than the actual distance, sometimes much greater.

It is possible to build good observation models—conditional probability functions relating RTT measurement to actual distance—using large numbers of measurements in the environment of interest [27,28]. As stated above, the RTT measurements should *not* be treated directly as distance measurements. The resulting observation models can then be used in various “filtering” approaches to localization, such as Bayesian grid update [27,28,32].

Two-sided RTT (as per IEEE 802.11-2016) requires the cooperation of the APs and, as such, has a scaling issue in that the APs may become overloaded if a large number of smart phones are simultaneously trying to determine their locations.

## 3. One-Sided FTM RTT

Currently, the fraction of APs that advertise (or implement) the IEEE 802.11-2016 protocol still remains limited, which creates a problem for indoor localization. One response to this is the use of a simpler protocol that does *not* require cooperation of the AP, but just measures the difference between the time of sending a message and the time of receiving an acknowledgment. The advantage of this approach is that it can be used with most Wi-Fi APs, including those not implementing IEEE 802.11-2016. That is, the AP does not have to cooperate; it only does what it normally does, which is send an acknowledgment. Another advantage is the much reduced load on the AP that would otherwise result when many users try to determine their location at the same time.

One disadvantage of one-sided RTT is that the “turn-around” time in the AP—which needs to be subtracted out—is initially not known. It ought to be about equal to the “short inter frame space” (SIFS), which is 16 μs for most Wi-Fi standards of interest here (IEEE 802.11n, 802.11ac 5 GHz, 802.11ax)—and 10 μs for some older standards (IEEE 802.11n 2.4 GHz, 802.11g, 802.11b, 802.11-1997 DSSS). That corresponds to 2400 m (one-way) for most Wi-Fi standards (and 1500 m for some older standards). However, this estimate is not to be relied upon, since the actual offsets often are larger, more like 2600 to 2700 m. The turn-around time needs to be known accurately, since it is by far the largest component of the time between sending and receiving an acknowledgment.

The FTM RTT API in Android 12—which was released at the end of 2021—has methods suitable for one-sided RTT (**RangingRequest.Builder.addAccessPoint()** and **RangingRequest.Builder.addNon80211mcCapableAccessPoint()**). One can choose the number of trials in a “burst” and obtain the average of the RTT of those exchanges that were successful, as well as their standard deviation. For *N* messages in the burst, there will be *N* measurements (unlike the situation with two-sided RTT mentioned above, where only (N−1) measurements are available).

## 4. What Is New?

In this paper, we present:

- The use of FTM RTT with Wi-Fi APs that do *not* support the IEEE 802.11-2016 protocol—including “legacy” APs;
- Observation models for one-sided FTM RTT in cluttered indoor environments—where a clear LOS is rare;
- The Bayesian grid update methods for indoor localization using one-sided RTT;
- Methods for determining the bias/offset of one-sided RTT with respect to particular AP types;
- The ability to use the same observation model in a quite different context, without adjustment.

## 5. “Filtering” Raw RTT Measurements

Because RTT measurements are subject to various kinds of errors, methods for making use of a history of measurements can be used to improve localization. Results can be improved using “filtering” methods such as Kalman filters, particle filters [12], or Bayesian grid update methods [32]. Some of these are based on assumptions about probability distributions, such as assuming a Gaussian shape, or at least being parameterized in some simple fashion. Other methods can take into account prior information or constrain the results based on prior knowledge of the environment.

The probability distributions encountered in RTT tend not be symmetrical and have long tails, and so, do not fit the Gaussian model. The Bayesian grid update method has the advantage of not requiring a parameterized representation and is also able to incorporate prior information. It does require an observation model, which relates the measurement to the ground truth.

## 6. Observation Model and Transition Model

The observation model is a conditional probability distribution p(y|x) of reported RTT measurement *y* given the actual distance *x*. This is used with the Bayes rule to update probabilities on a grid of possible locations of the initiator. For speedy computation, given *y*, an intermediate data structure, the “rate vector” $r_{y}\left(x\right)$, is first computed. In detail, for a reported RTT value $y_{i}$ for access point AP_i_, say, one pre-computes $r_{y_{i}}\left(x_{k}\right)=p\left(y_{i}|x_{k}\right)$ for a set $\left\{x_{k}\right\}$ of equispaced actual distances. The Bayesian grid update algorithm then simply steps through each cell of the grid and multiplies the probability there by $r_{y_{j}}\left(x_{i,j}\right)$ using the known distance ($x_{i,j}$) between the grid cell *j* and the access point AP_i_. The needed value is interpolated from entries in the discretized rate vector $r_{y_{i}}$. This operation is repeated for each AP that responded.

The transition model deals with the motion of the initiator between RTT updates. In the absence of any specific information, this can be taken to be a random walk with a standard deviation based on a comfortable walking speed. The transition model has little effect if the distance traveled in the time taken to update the RTT values is small relative to the spacing of the underlying grid. For example, if the update rate is 4 Hz and the walking speed is 1 m/s (as in the examples here), then the initiator only moves 0.25 m between RTT updates. Therefore, the spread of the probability distribution induced on a grid with 0.5 m spacing by the transition model is small. Information from device accelerometers and gyroscopes, if available, could be used to provide a more accurate transition model. We focus here, however, on what can be achieved based only on RTT measurements, without “sensor fusion” with other information.

Note that the Bayesian grid may contain prior information, such as walls through which it is known the initiator cannot pass. This can help constrain the solution. For additional details on the Bayesian grid update method, see [27,28,32].

## 7. Building an Observation Model from Measurements

**Figure 1:**
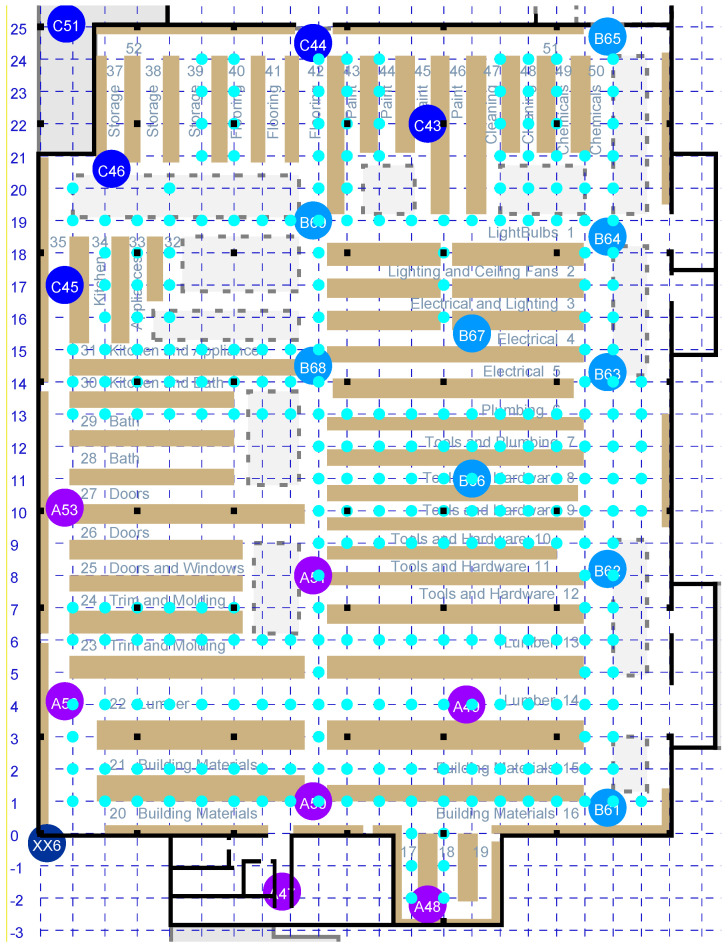
Floor plan of a box store with 20 APs marked with large colored circles. The main floor area is about 82 m by 107 m. Beige rectangles denote shelves filled with merchandise. Cyan-colored dots indicate 278 points with known coordinates that are accessible (e.g., not under shelves). These are at the intersections of lines in the concrete floor, indicated using dashed lines.

Signal propagation and its influence on measured RTT depend on the environment. It is best to base an observation model on measurements in an environment similar to that where the system is to be deployed. Previously, measurements in single- and multi-story homes led to the “double-exponential” observation model [27,28]. With the expectation that this model (designed for two-sided RTT) might, unaltered, not work as well in the case of one-sided RTT, experiments were carried out to investigate one-sided RTT in a large box store—which has shelves stocked with merchandise separating aisles in which the initiator may be located (see Figure 1). In this situation, there rarely is a clear LOS between an initiator and a responder, and signals are slowed down (and hence, delayed) as they pass through the merchandise stacked on the shelves.

The needed conditional probability distribution can be estimated experimentally by taking many measurements of RTT with known distances. First, since the locations of the *N* APs need to be known anyway, one can easily obtain RTT values for all of them from each AP location. These yield N(N−1) measurements. However, this is typically not enough to get a clear idea of what the conditional probability distribution is. Obtaining more samples can be a tedious operation, well worth automating, unless a grid of points with known locations happens to be marked already. This is the case, for example, when a set of parallel lines intersects a second set of parallel lines at right angles on the floor (see the cyan dots in Figure 1). RTT measurements of all APs within range were collected from each of the accessible grid points.

**Figure 2:**
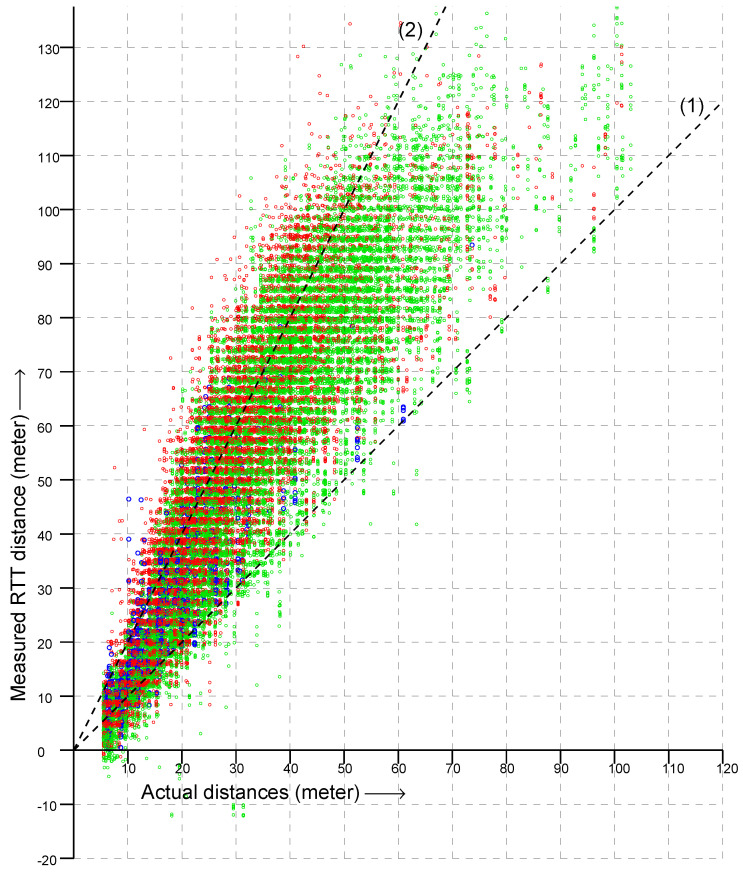
Scattergram of over 32,000 RTT measurements (vertical axis) versus actual distance (horizontal axis). (Green, measurements in the 2.4 GHz band; red, in the lower and upper 5 GHz band; blue, in the DFS part of the 5 GHz band). Dashed lines indicate slope (1) and (2).

In Figure 2 are shown a bit over 32,000 RTT measurements to 20 APs from 278 known locations on the floor of a large box store (measurements from each location were taken on more than one occasion, and each AP advertises multiple BSSID on the same frequency). The dashed lines in the figure have slopes 1 and 2. If RTT always gave the actual distance, all points would be on the line of slope 1. Instead, most RTT measurements were between the actual distance and twice the actual distance—with some measurements even larger.

**Figure 3:**
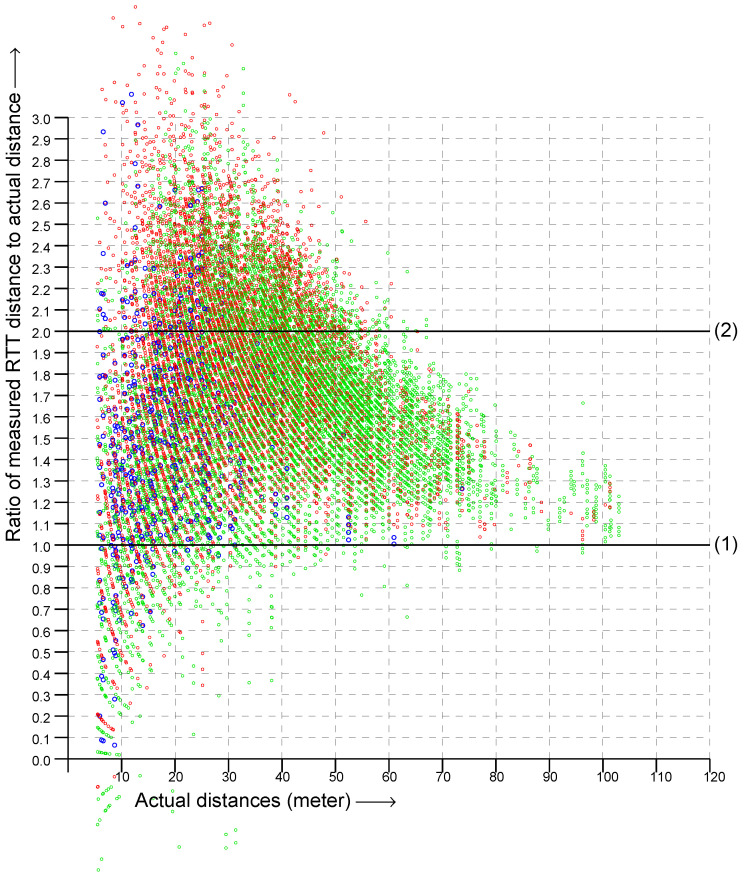
Scattergram of the ratio of RTT measurements to actual distance (vertical axis) versus actual distance (horizontal axis). (Green, measurements in the 2.4 GHz band; red, in the lower and upper 5 GHz band; blue, in the DFS part of the 5 GHz band). Solid lines mark ratios (1) and (2).

**Figure 4:**
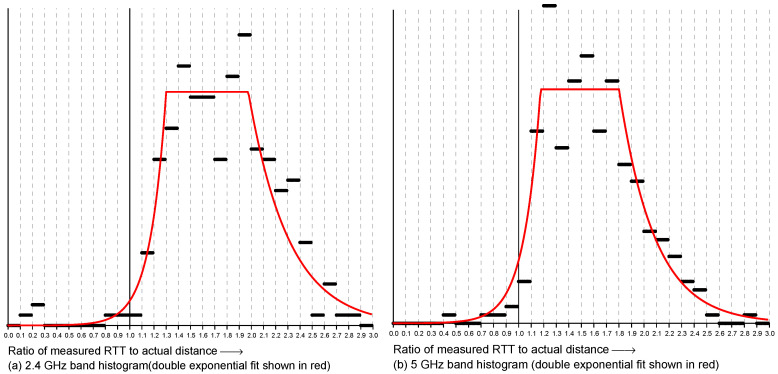
Histograms of ratios of RTT measurements to actual distances. Most measurements are larger than the actual distance, some twice as large or more: (**a**) 2.4 GHz band and (**b**) 5 GHz band. Coarse double-exponential fits shown in red.

Overall, the vertical spread in the scattergram became larger as the actual distance became greater (i.e., going to the right in the scattergram). This suggests plotting the *ratio* of RTT measurement to the actual distance instead in order to simplify the problem of fitting a reasonable observation model. This is shown in Figure 3. Next, to further simplify the problem, we can try and collapse the two-dimensional distribution into one dimension by summing over the actual distance to obtain a histogram of the ratio of measured to actual distance. The result is shown in Figure 4. This looks somewhat similar to the “double-exponential” observation model of one-story and multi-story homes [27,28]—with one big difference, which is that the peak is *much* broader with one-sided RTT than with two-sided RTT.

For two-sided RTT, most of the measurements fall within 1.07 and 1.17 of the true distance [27], while with one-sided RTT most measurements fall within about 1.1- and 2.2-times the true distance—which presents a much bigger problem for estimating the initiator position. Surprisingly, despite this, good localization results can be obtained using a double-exponential fit based on this histogram. (Note, by the way, that a Gaussian would not be a good fit here because the histogram is not symmetrical and because the tails decay too slowly).

However, Figure 3 shows that the vertical spread in the ratio is *not* nearly constant, but instead, goes down with actual distance. The reason for the decrease in spread is that the signal becomes weaker with distance, and if it is too corrupted by passing through various materials, it will no longer produce a useful response for RTT measurement. Therefore, as distance increases, we see fewer and fewer signals that have been substantially slowed down on their way by passing through multiple layers of various materials. Further, signals that are reflected by something away from the LOS will be subject to smaller fractional changes of their total path length as the actual distance increases. Therefore, overall, fewer signals with an apparent distance much greater than the actual distance are seen as the distance increases. This suggests that we should be able to obtain a more fine-tuned observation model by fitting a function directly to the original scattergram.

**Figure 5:**
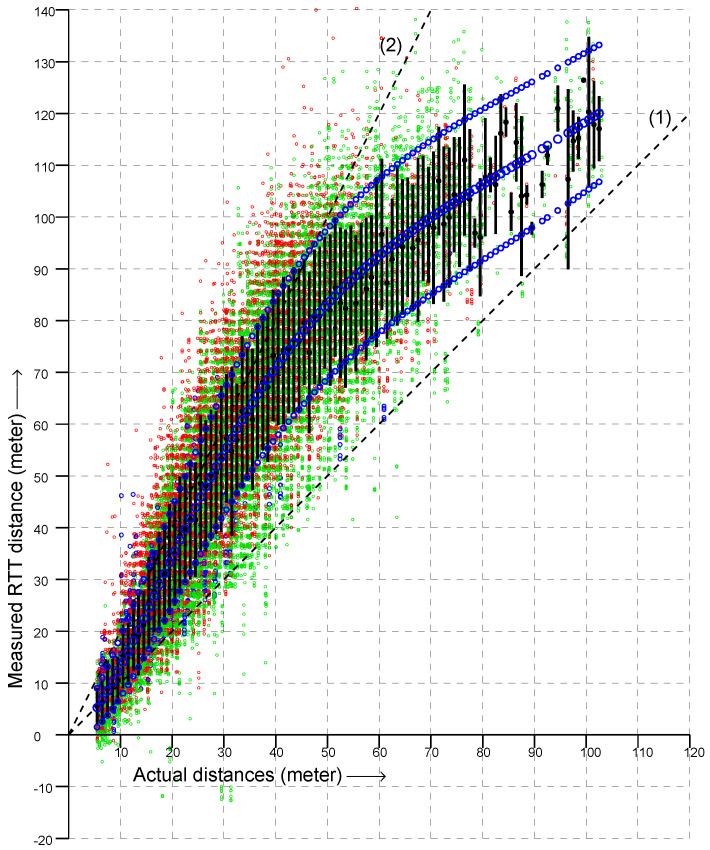
Scattergram with curve fit superimposed. Dashed lines indicate slope (1) and (2). Each vertical black bar is a line from μ−σ to μ+σ where μ and σ are the average and standard deviations of the RTT response for that actual distance. The corresponding values for a curve fit are shown as small blue circles.

A good fit can be obtained by inspection of the curves of the averages and standard deviations in Figure 5. For the average of RTT measurements corresponding to actual distance *x*, we can use the approximation

(2) $$\mu \left(x\right)=x\left(1+A\alpha \left(x-x_{0}\right)e^{-\alpha (x-x_{0})}\right)$$

where $x_{0}$ is the distance of the closest approach between the initiator and responder and *A* and α are the parameters of the fit.

From the data in the scattergram, we find $x_{0}=5.5$ m (the APs are mounted 6.5 m off the ground), $\alpha =1/(x_{m}-x_{0})=0.043$, and $A=e(f\left(x_{m}\right)-1)=2.23$, where $x_{m}=30$ m is the actual distance at which the average ratio deviates most from 1, while $f(x_{m})=1.82$ is the peak of that ratio.

For the standard deviation, we can use the estimate

(3) $$\sigma \left(x\right)=\left(\sigma_{0}+m\left(x-x_{0}\right)\right)e^{-\beta (x-x_{0})}$$

where $\sigma_{0}$ is the offset and $\sigma_{m}$ the slope of a linear fit, with the exponential term modeling decay with actual distance.

From the data in the scattergram, we find $\sigma_{0}=4$, $\sigma_{m}=0.55$, and β=1/λ=0.015, where λ=66 m is the “half-life” of decay of the standard deviation curve. Finally,

(4) $$p\left(y|x\right)=\frac{1}{\sqrt{2\pi }\sigma \left(x\right)}e^{-\frac{1}{2}{\left((y-\mu (x))/\sigma (x)\right)}^{2}}$$

Importantly, exact details of this somewhat *ad hoc*-looking functional fit are not very important since the Bayesian grid update methods appear to work well with an approximate observation model (see also Section 12).

**Figure 6:**
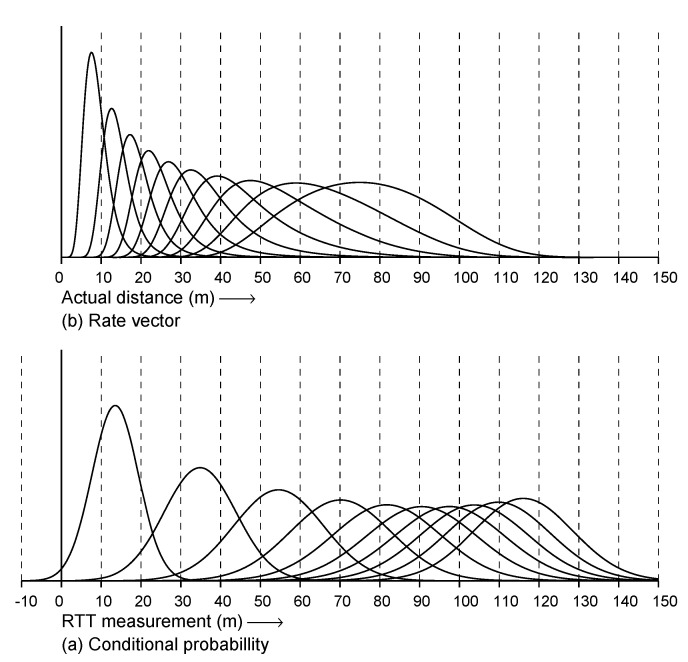
Fitted observation model. (**a**) Conditional probability plotted versus RTT measurement. The curves are for (left to right) actual distances of 10 m, 20 m, …, 100 m. (**b**) Rate vectors plotted versus actual distance. The curves are for (left to right) RTT measurements of 10 m, 20 m, …, 100 m.

Figure 6 shows the fitted observation model in two different ways: (a) conditional probability given RTT measurement and (b) rate vector versus actual distance. The latter is used in the Bayesian grid update step. As the width of the curves in the latter increase as the RTT measurements become large (and the peaks become lower), it is clear that smaller RTT measurements constrain the probability distribution more tightly than large RTT measurements. Therefore, there is an automatic “weighting” that emphasizes the information from nearby APs, yet does not ignore that from APs further away.

## 8. One-Sided FTM RTT Offset

One difference between one-sided and two-sided RTT is that the former has a very large offset, the order of 2400 to 2700 m (the value of which depends on the combinations of the initiator and responder). (A few APs have smaller, non-standard offsets, in the range 400–500 m).

The offset could be estimated by measuring the RTT distance from a location at a known distance from the AP. Such a measurement from a single fixed position, however, does not produce an accurate result because of the so-called “position-dependent” error [27,28]. The average offset can instead be estimated accurately using an Android app such as **WifiRttScanX** [33] or **WifiRttScan** [34] from measurements taken at a *series* of locations.

We found that this offset, while different for different Wi-Fi AP models, was typically the same for APs of a particular model. The “organizationally unique identifier” (OUI) is a 24 bit number that uniquely identifies a vendor or manufacturer. It forms the high order half of the 48 bit MAC address (BSSID). As such, it can be obtained without physical access to the AP. Often, a large Wi-Fi installation will use APs that all have the same OUI or perhaps just a few different OUIs. (In the large box store example here, all the APs in the interior of the building had the same OUI, while APs added later in the garden section, and those added outside the building, had different OUIs, with quite different offsets). (Note, however, that in some cases, a manufacturer may switch Wi-Fi chipsets without changing the model number of a device and without changing the OUI, leading to different offsets for APs sharing the same OUI—but this approach appears to be rare).

## 9. Selecting Which APs to Range to

It takes some time to range to an AP, so there is a tradeoff between ranging infrequently to many APs versus ranging more often to fewer APs. The time to range per AP depends on the initiator and the responder, as well as the number of “tries” in a burst and other factors (e.g., about 25 ms for a burst of eight tries on Google Pixel 5 versus Google Wi-Fi). If one ranges to ten or fewer APs, one should be able to maintain about a 4 Hz refresh rate (conveniently, Android allows up to 10 APs in a single **RangingRequest**).

In an installation with many APs, some strategy must be developed to pick the APs thought most likely to provide useful localization information at any given time. In our example, there are 20 APs inside the main building, and ranging to them is carried out in both the 2.4 GHz band and the 5 GHz band, leading to a potential 40 BSSIDs to consider. (Although, currently, APs that happen to operate in the dynamic frequency selection (DFS) part of the 5 GHZ band cannot be used, which cuts the total by about a third). Here are three of the strategies that we tried:

(1) First: distance. Distant APs may not respond at all or, if they do, contribute little to resolving ambiguity, as we saw that the “rate vector” curves become wide for large actual distances (Figure 6b). Therefore, one simple approach is to select the APs that are nearest to the current estimate of the position of the initiator. This approach requires sorting APs by distance when a ranging request is built. The distances have to be estimated after the Bayesian grid is adjusted (using either the most likely or the expected value of the initiator position based on the current probability distribution on the grid).

(2) Second: signal strength. We can pick the APs with the highest signal strength. The ranging responses do include signal strength, but of course, only for the APs in the current ranging request. Therefore, periodic Wi-Fi scans are needed to detect APs that have come into range (and to make a note of those that are now too far away). On Android this can be performed without interfering with the ranging process, despite the fact that a full Wi-Fi scan may take between 2.5 and 3.5 s.

The signal strengths in the Wi-Fi scan and in the ranging response differ. One reason is that the Wi-Fi scan is based on the 20 MHz bandwidth used for the beacons, while ranging may be requested on a 20, 40, or 80 MHz bandwidth. The signal strength from ranging is reported several times a second, whereas Wi-Fi scans take much longer. One has to decide then which of the two signal strength values to use. After some experimentation, we chose to use the Wi-Fi scan signal strength as being more consistent.

(3) Finally: time last seen. One may wish to prioritize APs that have been “seen” recently (either in a Wi-Fi scan or while ranging). Note that APs that are not picked up in a Wi-Fi scan should *not* be immediately considered to have gone out of range, since Wi-Fi scans are on a “best effort” basis and not guaranteed to pick up each AP within range every time. Rather, a “keep alive” period can be used to keep them under consideration for some time after they were last “seen.”

We experimented with these approaches—using estimated distance, using signal strength, and using time since last seen—when deciding which APs to request ranges for. One might think that the use of the distance as a criterion should be ideal, but the problem is that some APs, while nearby, may be hidden behind much material and not provide any ranging response, or not accurate ones. All three strategies work, but the sorting on “time last seen” appears to work best.

Typically, each AP announces multiple BSSIDs (with different SSIDs) in the beacon (in our case, eight of them in the 5 GHz band and seven in the 2.4 GHz band). One might think there would be some advantage to ranging to all of these. Not surprisingly, however, the ones operating on the same frequency tend to produce *very* similar RTT results. Therefore, there is less useful new information provided when ranging to a second BSSID on the *same* AP than when ranging to a *different* AP. However, ranging results in the 2.4 GHz and 5 GHz bands *are* different [27], and so, the number of useful measurements can be doubled by using one BSSID in each of the two bands. In our case, this meant that we potentially had 40 ranging targets, rather than just 20.

The offsets in the two bands are very different and need to be determined separately (this is in part because signal propagation and fading will be different in the two bands). In addition, the offsets may also depend on the frequency *within* a band. For example, the offset in the DFS part of the 5 GHz band (5.26, …, 5.32 and 5.50, …, 5.64 GHz) may be different from the offset in the lower 5 GHz band (5.18, …, 5.24 GHz) and in the upper 5 GHz band (5.745, …, 5.885 GHz).

This means that the frequency on which the AP operates must be known. Wi-Fi scans are needed, since the frequency assignments of APs in a large installation typically are rearranged periodically.

Related to the above is the question of how many trials to use in a burst. In the current API, a burst can contain from 2 to 31 trials, and the mean and standard deviation of the results (but not the individual measurements) are provided by the API. The advantage of longer bursts is limited by the dominance of the “position-dependent” error over ordinary measurement error [27,28]. Long bursts slow down the update rate. In our experiments, other factors dictated that we limit bursts to just two trials (see the next section).

## 10. Sample Screenshot

**Figure 7:**
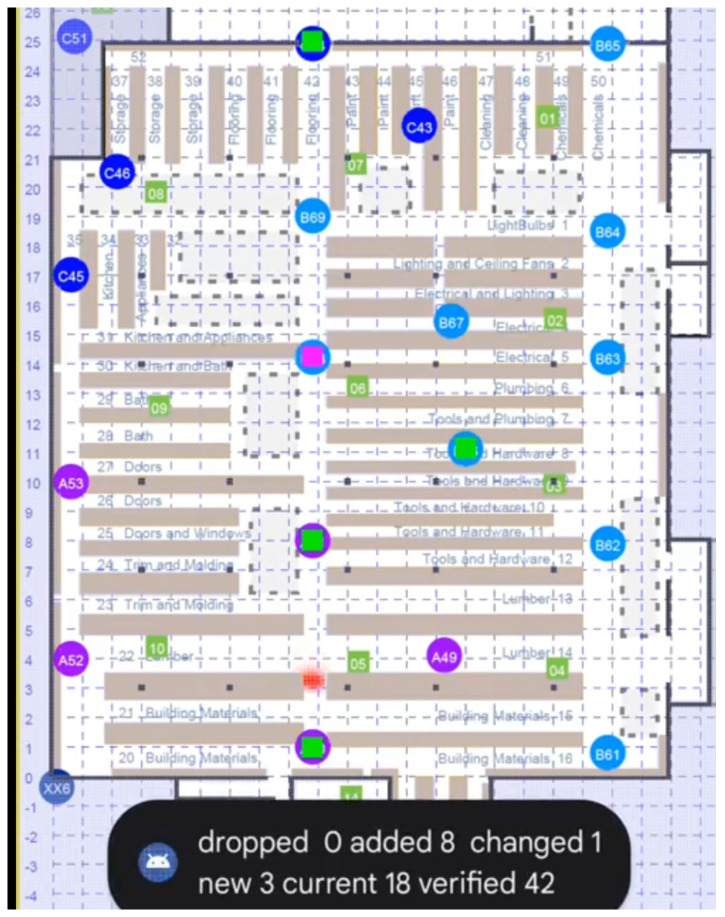
Screenshot of the FTMRTT application using Bayesian grid update method as a “heat map”. The “hot spot” (red) is the area of high probability. APs currently responding to ranging requests are shown as green squares. APs in the current selection *not* responding are shown as magenta squares. The cells in the Bayesian grid here are 0.5 m on a side. The Bayesian grid update method uses the observation model developed here.

Figure 7 is a screenshot taken from a video recording of the Android app running inside a large box store [35]. Some number of APs are interrogated at any given time (ten at most). The ones that respond to ranging requests are shown in green, while those not responding are shown in magenta. The red “hot spot” would be almost always in the correct aisle and rarely more than 3–4 m from the correct position. It tended to lag perhaps 1–2 m behind the position of the initiator, in part because of a rather simplistic transition model (all “filtering” methods obtained better results by lagging behind a bit).

Accuracy was limited in part by the fact that all APs here were set to use only the 20 MHz bandwidth, even when operating in the 5 GHz band (accuracy is approximately inversely proportional to bandwidth). Furthermore, the APs set to operate in the DFS portion of the 5 GHz band could not be used. This reduced the number of available APs by about a third.

Finally, in the first release of the new API, it was best to work with bursts of just *two* tries, because longer bursts would fail if a single measurement in the burst failed. Naturally, more accurate results can be expected if the results of more than two attempts can be averaged.

## 11. Some Comparisons

Raw distance measurements using one-sided RTT are less accurate than those obtained using two-sided RTT (the quality depending on the responding AP). The ability to range to more APs using the one-sided approach (no longer restricted to only those supporting the IEEE 802.11-2016 protocol) can compensate for this. Still, the 3–4 m accuracy attained here for one-sided RTT with the 20 MHz bandwidth was not as good as the 1–2 m accuracy attained with two-sided RTT with the 80 MHz bandwidth [15,27,28]. Working with APs with a higher bandwidth can improve matters (most now support 80 MHz).

Under favorable circumstances, “fingerprinting” RSSI may be able to attain such accuracies, but requires preparatory work that does not scale well and has to be repeated when large objects in the environment are moved (whereas with RTT—one-sided or two-sided—all we need to know are the locations of the APs).

Another great advantage of RTT (one-sided and two-sided) is that no new hardware or special beacons are required. Wi-Fi APs are already installed everywhere, and ordinary cell phones can communicate with them. Further, privacy concerns are addressed in that the responder (AP) does *not* have enough information to compute the distance to the user, only the “initiator” (cell phone) has all of the timestamps needed. Several competing technologies fall short in these regards. For example, “angle-of-arrival” methods require specialized APs with many antennas, and the distance is computed in this specialized device rather than in the cell phone.

The accuracy of ranging methods is inversely proportional to the bandwidth. Thus ultra-wide-band (UWB) technology would appear to provide a distinct advantage. UWB, however, is ham-strung by the requirement that only very low power can be used, which limits its range, as well as the need for specialized hardware not available in typical cell phones.

As noted before (see, e.g., Section C in [27]), channel state information (CSI) could potentially provide more information for accurately estimating the time of arrival, but is currently not available in the API of cell phones.

Meaningful comparisons of accuracy are difficult to make if the environments are different, the layout of APs is different, or the density of APs is different. In particular, with a high-enough density of APs, the indoor localization problem becomes trivial. As an extreme case, consider a set of APs arranged in a 1 m grid. In this case, the cell phone is never more than 0.707 m from the nearest AP. More generally, for a square grid with density ρ APs per unit area, even a simple-minded assignment of position to the nearest AP has an error less than

(5) $$\epsilon_{\max}=1/\sqrt{2\rho }$$

(the rms error is even less). Naturally, less uniform layouts will have larger position errors. In any case, a high AP density can make the localization problem easier.

Therefore, in evaluating the quality of indoor localization methods and comparing them, it is important to take note of the density of responders (or other required artifacts, such as beacons). The tests reported here were at a relatively low density (20 APs per 9000 m^2^), with a non-uniform layout.

## 12. Test of One-Sided RTT in a Different Environment

We wondered how sensitive the method was to the details of the observation model, that is: Does one always have to collect many measurements in a new environment and perform some careful curve fitting? To test this, we tried the system in a very different setting—an outdoor situation in a multi-building hotel/condo complex. Here, all the APs are behind walls, so there are *no* line-of-sight (LOS) paths, and much of the space is clear (aside from trees and other landscaping). The space is not cluttered with merchandise as in the “large box store”. Further, GPS could provide some crude ground truth (even though the accuracy of GPS in smart phones is limited).

**Figure 8:**
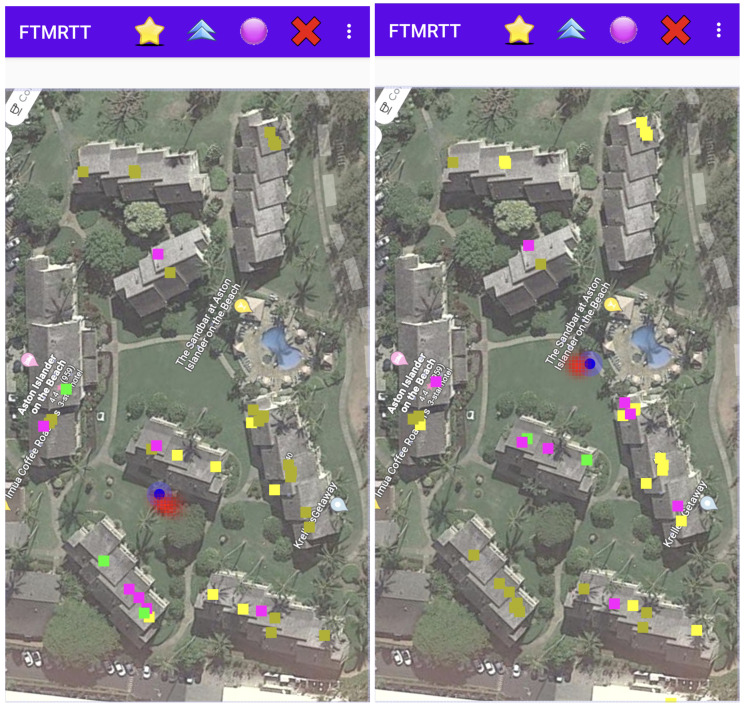
Two sample screenshots of the FTMRTT application using the Bayesian grid update method as a “heat map”. The “hot spot” (red) is the area of high probability. The blue spot is the GPS estimate. APs currently responding to ranging requests are shown as green squares. APs in the current selection *not* responding are shown in magenta. APs not in the current selection, but whose frequency and bandwidth are known are shown in yellow.

Figure 8 shows two screenshots of the Android app running in the hotel/condo environment. As before, APs currently responding are shown in green, those ranged to, but not responding in magenta, and those not in the current selection, but whose frequencies and bandwidths are known (from Wi-Fi scans) in yellow.

Note that the density of APs in this example is even lower, about 25 APs (working in both the 2.4 GHz band and in the 5 GHz band) in an area of about 108 m × 182 m (i.e., about twice as large as the big box store). Note also that the APs are not uniformly distributed, but instead concentrated near the mid-lines of eight elongated buildings.

The method works even in this very different environment—and actually, is competitive in accuracy with cell phone GPS—except in the upper right where the density of known APs is particularly low.

## 13. Conclusions

In this paper, we presented:

- The use of FTM RTT with Wi-Fi APs that do *not* support the IEEE 802.11-2016 protocol—including “legacy” APs;
- Observation models for one-sided FTM RTT in cluttered indoor environment—where a clear LOS is rare;
- The Bayesian grid update methods for indoor localization using one-sided RTT;
- The Methods for determining the bias/offset of one-sided RTT with respect to to particular AP types;
- The ability to use an observation model in a quite different context without adjustment.

## 14. Future Work

While the startup effort for indoor localization using existing APs is much less than that required for some competing approaches (such as “fingerprinting” of signal strength or “learning”), there remains some work in determining the locations of the APs relative to the floor plan. This information may be available from the entity that set up the Wi-Fi system. An interesting question for future work is whether these positions can be recovered automatically, along with the offsets for each AP, using something analogous to simultaneous localization and mapping (SLAM) in robotics.

## Data Availability

Not applicable.
